# RNF144A exerts tumor suppressor function in breast cancer through targeting YY1 for proteasomal degradation to downregulate GMFG expression

**DOI:** 10.1007/s12032-021-01631-6

**Published:** 2022-02-01

**Authors:** Yin-Ling Zhang, Jin-Ling Cao, Ye Zhang, Li Liao, Ling Deng, Shao-Ying Yang, Shu-Yuan Hu, Yan Ning, Fang-Lin Zhang, Da-Qiang Li

**Affiliations:** 1 Fudan University Shanghai Cancer Center, Shanghai Key Laboratory of Medical Epigenetics, International Co-Laboratory of Medical Epigenetics and Metabolism of Ministry of Science and Technology, Institutes of Biomedical Sciences, Fudan University, Shanghai, 200032 China; 2 Cancer Institute, Shanghai Medical College, Fudan University, Shanghai, 200032 China; 3 Department of Pathology, Obstetrics and Gynecology Hospital, Fudan University, Shanghai, 200011 China; 4 Department of Breast Surgery, Shanghai Medical College, Fudan University, Shanghai, 200032 China; 5 Shanghai Key Laboratory of Breast Cancer, Shanghai Medical College, Fudan University, Shanghai, 200032 China; 6 Shanghai Key Laboratory of Radiation Oncology, Shanghai Medical College, Fudan University, Shanghai, 200032 China

**Keywords:** Breast cancer, RNF144A, Protein degradation, YY1, GMFG

## Abstract

Ring finger protein 144A (RNF144A), a poorly characterized member of the RING-in-between-RING family of E3 ubiquitin ligases, is an emerging tumor suppressor, but its underlying mechanism remains largely elusive. To address this issue, we used Affymetrix GeneChip Human Transcriptome Array 2.0 to profile gene expression in MDA-MB-231 cells stably expressing empty vector pCDH and Flag-RNF144A, and found that 128 genes were differentially expressed between pCDH- and RNF144A-expressing cells with fold change over 1.5. We further demonstrated that RNF144A negatively regulated the protein and mRNA levels of glial maturation factor γ (GMFG). Mechanistical investigations revealed that transcription factor YY1 transcriptionally activated GMFG expression, and RNF144A interacted with YY1 and promoted its ubiquitination-dependent degradation, thus blocking YY1-induced GMFG expression. Functional rescue assays showed that ectopic expression of RNF144A suppressed the proliferative, migratory, and invasive potential of breast cancer cells, and the noted effects were partially restored by re-expression of GMFG in RNF144A-overexpressing breast cancer cells. Collectively, these findings reveal that RNF144A negatively regulates GMFG expression by targeting YY1 for proteasomal degradation, thus inhibiting the proliferation, migration, and invasion of breast cancer cells.

**Supplementary Information:**

The online version contains supplementary material available at 10.1007/s12032-021-01631-6.

## Introduction

Protein posttranslational modification plays an important role in regulating the stability and activity of key-signaling proteins [1]. In particular, the ubiquitin–proteasome pathway is critically involved in maintaining cellular homeostasis of cytosol and nuclear proteins of eukaryotic cells [2]. This process is catalyzed by a ubiquitin-activating enzyme (E1), a ubiquitin-conjugating enzyme (E2), and various E3 ubiquitin ligases [3]. E3 ubiquitin ligases play a key role in determining substrate specificity and catalyzing the transfer of ubiquitin from E2 enzyme to its substrates [4]. The human genome encodes about 600 E3 ubiquitin ligases, which are divided into three main families based on their structure and catalytic mechanism, including RING (really interesting new gene), HECT (homologous to the E6AP carboxyl terminus), and RBR (RING-in-between-RING) [4, 5]. RBR-type E3 protein ligases are defined by the presence of a RING1 domain, followed by an ‘in-between RING’ (IBR) domain, and a RING2 domain [6, 7]. Unlike classic HECT- and RING-type E3 ligases, RBR-type E3 ligases use a RING/HECT hybrid-like mechanism to catalyze the transfer of ubiquitin to target proteins for proteosomal degradation [8–10].

Emerging evidence shows that RBR-type E3 ligases exert essential roles in neurodegenerative disease, infection, inflammation, and cancer [11]. Ring finger protein 144A (RNF144A) is a poorly characterized member of the RBR family of E3 ligases [6]. Limited information shows that RNF144A promotes apoptosis during DNA damage [12] and under oxidative stress [13]. Consistently, loss of RNF144A contributes to carcinogen-induced bladder tumorigenesis [14]. We recently demonstrated that RNF144A is epigenetically silencing in breast cancer cells by promoter hypermethylation [15], suppresses breast cancer progression through promoting the degradation of oncoprotein heat-shock protein family A member 2 (HSPA2) [16], and regulates the sensitivity of breast cancer cells to poly(ADP-ribose) polymerase (PARP) inhibitors through modulating the degradation PARP1 [17]. Together, these results indicate that RNF144A acts as an emerging tumor suppressor, but its underlying mechanisms still remain poorly understood.

Glia maturation factor-γ (GMFG) is a 16.8-kDa protein that was initially identified as a growth and differentiation factor acting on neurons as well as glia in the vertebrate brain [18]. Subsequent studies revealed that GMFG is preferentially expressed in proliferative, microvascular endothelial, and inflammatory cells [18, 19], and is an important regulator for angiogenic sprouting during angiogenesis in zebrafish [20]. In addition, GMFG regulates monocyte migration through modulation of β1-integrin [21], neutrophil chemotaxis by modulating actin cytoskeleton reorganization [22], and the migration and adherence of human T lymphocytes [22]. Bioinformatic analysis of GMFG in pan-cancers revealed that GMFG causes different survivals for different cancers through modulating tumor progression, immune response status, and tissue-specific tumor microenvironment [23]. High expression of GMFG is associated with colorectal cancer metastasis [24] and poor prognosis of patients with epithelial ovarian cancer [25], glioblastoma, lower grade glioma, lung squamous cell carcinoma, ocular melanomas, and prostate cancer [23]. Consequently, downregulation of GMFG suppresses colorectal cancer cell migration and invasion [24]. Despite these advances, the biological function and related regulatory mechanism of GMFG in breast cancer are unclear.

Transcription factor YY1 has been shown to regulate gene transcription by either binding to gene promoter or as a cofactor of a variety of transcription factors in multicellular organisms [26]. YY1 is overexpressed in variety types of human cancer including breast [27, 28], colon [29], lung [30], glioma [31], bladder [32], and prostate [33], and plays essential roles in cell proliferation, cell viability, epithelial-mesenchymal transition, metastasis and drug/immune resistance[34, 35]. In breast cancer, YY1 promotes the stemness [34], clonogenicity, migration, invasion, and tumor formation of breast cancer cells [28, 36–38]. In addition, YY1 contributes to endocrine resistance [39] and chemoresistance [34] of breast cancer cells. Emerging evidence shows that YY1 undergoes proteasome-dependent degradation in cancer and other cells [40–43]. However, the regulatory mechanisms for YY1 degradation and downstream target genes of YY1 in breast cancer cells are still largely unknown.

In this study, we found that YY1 transcriptionally activates GMFG expression, and RNF144A interacts with YY1 and promotes its degradation through the ubiquitin–proteasome pathway, thus blocking YY1-mediated induction of GMFG expression in breast cancer cells. Eventually, functional rescue assays demonstrated that the inhibitory effects of RNF144A on breast cancer cell proliferation, migration, and invasion was reversed by re-expression of GMFG in RNF144A-overexpressing cells. Together, these findings reveal a previously unrecognized role of the RNF144A-YY1-GMFG axis in breast cancer progression.

## Materials and methods

### Cell culture and reagents

Human breast cancer cell lines, MDA-MB-231, Hs578T, and MCF-7, and human embryonic kidney 293T (HEK293T) cell line were obtained from Cell Bank of Type Culture Collection of Chinese Academy of Sciences (Shanghai, China) and Shanghai Key Laboratory of Breast Cancer (Fudan University, Shanghai, China). All cell lines were authenticated by detection of mycoplasma, DNA-fingerprinting, cell viability, and endotoxin. Cells were cultured in high-glucose DMEM media (BasalMedia, #L110) supplemented with 10% fetal bovine serum (FBS; ExCell Biol, #FSP500) and 1% penicillin/streptomycin (BasalMedia, #S110B), and were maintained at 37 ^0^C in a humidified incubator with 5% CO_2_. MG-132 and cycloheximide (CHX) were obtained from Selleck (#S2619) and Cell Signaling Technology (#2112S), respectively. Other regents and chemicals were purchased from Sigma-Aldrich unless otherwise noted.

### Cell viability, colony-formation, migration, and invasion assays

Cell viability was determined using Cell Counting Kit-8 (CCK-8) kit (Yeasen, #40203ES92) according to the manufacturer’s instructions. In brief, cultured cells suspended in culture medium were seeded in 96‐well plates (1000 cells/well) in triplicate. In the following few day, 10 μl CCK-8 solution and 90 μl culture medium solution were added to each well. The plate was incubated for 2.5 h before measuring the absorbance at 450 nm wavelength using a microplate reader (SPECTROstar Nano, BMG Labtech). For colony-formation assays, cells were seeded in 6-well plates (1000 cells/well) in triplicate and cultured for 2–3 weeks. Survival colonies were photographed and counted after staining with 1% crystal violet. Transwell migration and invasion assays were performed using noncoated polycarbonate transwell inserts with 8-mm pore (Corning Falcon, #353097) and Matrigel Invasion Chambers (Corning, #354480), respectively. In brief, 5 × 10^4^ cells in 200 μl of serum-free medium were seeded in the top chamber, and 800 μl of growth medium containing 10% FBS was added into the lower chambers as a chemoattractant. After 24 h of incubation, cells were fixed and stained with 1% crystal violet, and migrated and invaded cells were counted under a microscope.

### Plasmid transfection and lentiviral infection

Flag-RNF144A, V5-ubiquitin, short hairpin RNA-targeting RNF144A (shRNF144A), and corresponding control constructs have been described previously [16]. To generate HA-YY1 and HA-GMFG constructs, YY1 and GMFG cDNAs were amplified by PCR and then subcloned into the lentiviral vector pLVX-IRES-Neo (Biofeng, #632181). The primers used for molecular cloning are provided in Supplementary Tables S1. DNA sequence was verified by DNA sequencing (HuaGene Biotech, Shanghai, China).

Transient plasmid transfection was performed using Neofect DNA transfection reagent (TengyiBio, #TF20120) according to the manufacturer's instructions. For generating stable cell lines expressing cDNAs or short hairpin RNA (shRNAs), HEK293T cells were transfected with each lentivirus expression vector and packaging plasmid mix using Neofect DNA transfection reagents. After 48 h of transfection, the supernatant containing viruses was collected, filtered, and used for infecting target cells in the presence of 8 mg/mL of polybrene (Sigma, #9268). Cells were cultured in the presence of 2 mg/mL of puromycin (Sangon Biotech, #A610593) or 5 mg/mL of G418 (Sangon Biotech, #A600958-0005) for 1 to 2 weeks.

### siRNA transfection

Three independent small interfering RNAs targeting YY1 (siYY1 #1-3) and corresponding negative control siRNA (siNC) were purchased from GenePharma (Shanghai, China). The siRNA target sequences are listed in Supplementary Table S2. The siRNA duplexes were transfected into cells using Lipofectamine 2000 transfection reagents (Invitrogen, #2041726) following the manufacturer's instructions. Knockdown efficiency was validated by immunoblotting after 48 h of transfection.

### Quantitative real-time PCR

Total RNA was extracted from cultured cells using TRIzol reagent (Invitrogen, #15596018) according to the manufacturer’s protocol, and then converted to cDNA using PrimeScript RT Master Mix (Takara, #RR036A). Quantitative real-time PCR (qPCR) assays were performed using SYBR Premix Ex Taq (Takara, #RR420) on an Eppendorf Mastercyclerep realplex4 instrument (Eppendorf). The PCR reaction program was consisted of a denaturation procedure at 95 °C for 95 s, followed by 40 cycles of 95 °C for 15 s, 55 °C for 15 s, and 72 °C for 45 s, and a final incubation procedure at 72 °C for 10 min. Expression levels of target genes were normalized to that of GAPDH. The primer sequences are provided in Supplementary Table S3.

### Dual luciferase reporter assays

The promoter sequence for 2000 bp upstream of transcription start site of GMFG was cloned into the pLG3-basic vector. Then, 1 μg pGL3-GMFG or control vector was transfected into cells using Neofect DNA transfection reagents. After 48 h of transfection, the activity of GMFG promoter was determined using the dual-luciferase reporter assay system (Promega, #E1910) according to the manufacturer’s protocol. All assays were repeated at least three times.

### Immunoblotting assays

Cells were collected after being washed with phosphate-buffered saline (PBS) for 3 times and lysed in RIPA lysis buffer containing 150 mM NaCl, 10 mM Tris (pH 7.2), 0.1% SDS, 1.0% Triton X-100, 1% deoxycholate, and 5 mM EDTA. Protein concentrations were determined using BCA protein quantification kit (Yeasen, #20201ES90). Cellular lysates were mixed with 4 × SDS-PAGE protein loading buffer, boiled at 100 °C for 5 min, separated on SDS-PAGE gels, and then transferred onto PVDF membranes (Millipore, #IPVH00010). The membranes were then blocked with 5% BSA for 2 h and then incubated with corresponding primary antibodies at 4 °C overnight, followed by incubation with secondary antibodies according to standard protocol. Rabbit polyclonal anti-RNF144A antibody was purchased from Lifespan (#LS-C162648). Rabbit polyclonal antibodies against GMFG (#13625-1-AP) and YY1 (#66281-1-Ig) were obtained from Proteintech. Mouse monoclonal anti-Flag (#F1804-1MG) and anti-Vinculin (#V9131) were obtained from Sigma-Aldrich. Rabbit monoclonal anti-HA antibody was purchased from Cell Signaling Technology (#3724).

### In vivo ubiquitination and cycloheximade (CHX) assays

In vivo ubiquitination and cycloheximade (CHX) assays have been described previously [16]. For examining ubiquitination levels of YY1, cells were transfected with expression plasmids encoding HA-YY1, V5-ubiquitin, and Flag-RNF144A alone or in combination. After 36 h of transfection, cells were treated with 10 µM MG-132 for the indicated times and then total cellular lysates were subjected to IP and immunoblotting analyses with the indicated antibodies as described previously in details [16]. For CHX chase assays, cells were treated with or without 100 μg/ml CHX for the indicated times and then subjected to immunoblotting with the indicated antibodies.

### Statistical analysis

All data are presented as mean ± SD from at least three independent experiments. Student’s *t* test was used to assess the difference for two groups, and *P* ≤ 0.05 was considered statistically significant.

## Results

### Identification of target genes of RNF144A

**Fig. 1:**
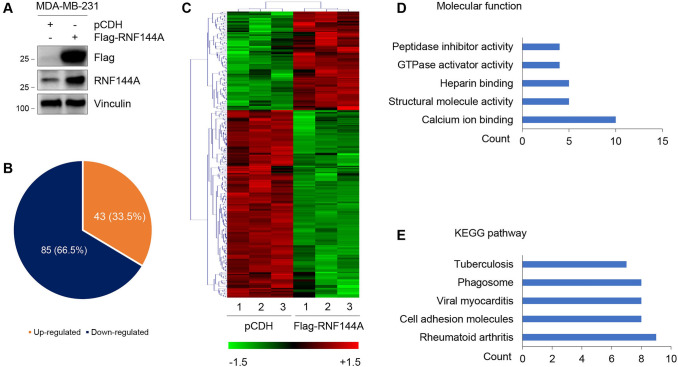
Identification of target genes of RNF144A. **A** Validation of MDA-MB-231 cell lines stably expressing pCDH and Flag-RNF144A by immunoblotting analysis. **B** Gene expression profiling using Affymetrix GeneChip Human Transcriptome Array (HTA2.0) in RNF144A-overexpressing MDA-MB-231 cells and control cells. **C** Clustering analysis of 128 differentially expressed genes between pCDH- and Flag-RNF144A expressing cells. **D** Gene ontology (GO) analysis of the molecular functions of 128 differentially expressed genes between pCDH- and Flag-RNF144A expressing cells. **E** KEGG pathway analysis of the enriched pathways of 128 differentially expressed genes between pCDH- and Flag-RNF144A expressing cells

To gain mechanistic insights into the biological functions of RNF144A in breast cancer progression, we established MDA-MB-231 cell lines stably expressing empty vector pCDH and Flag-RNF144A by lentiviral infection. The expression status of RNF144A in resultant cell lines was verified by immunoblotting assays (Fig. 1A). Gene expression profiling using Affymetrix GeneChip Human Transcriptome Array (HTA2.0) revealed that 128 genes were differentially expressed between RNF144A-overexpressing cells and control cells with a fold-change threshold of ≥ 1.5. Among them, 43 genes were upregulated, whereas 85 genes were downregulated, in RNF144A-overexpressing cells compared to control cells (Fig. 1B, C and Supplementary Table S4). Gene ontology (GO) analysis revealed that the top 5 molecular functions of 128 differentially expressed genes were involved in calcium ion binding, structural molecule activity, heparin binding, GTPase activator activity, and peptidase inhibitor activity (Fig. 1D). Kyoto Encyclopedia of Genes and Genomes (KEGG) pathway analysis showed that the top 5 enriched pathways of 128 differentially expressed genes were rheumatoid arthritis, cell adhesion molecules, viral myocarditis, phagosome, and tuberculosis (Fig. 1E).

### RNF144A negatively regulates GMFG expression

**Fig. 2:**
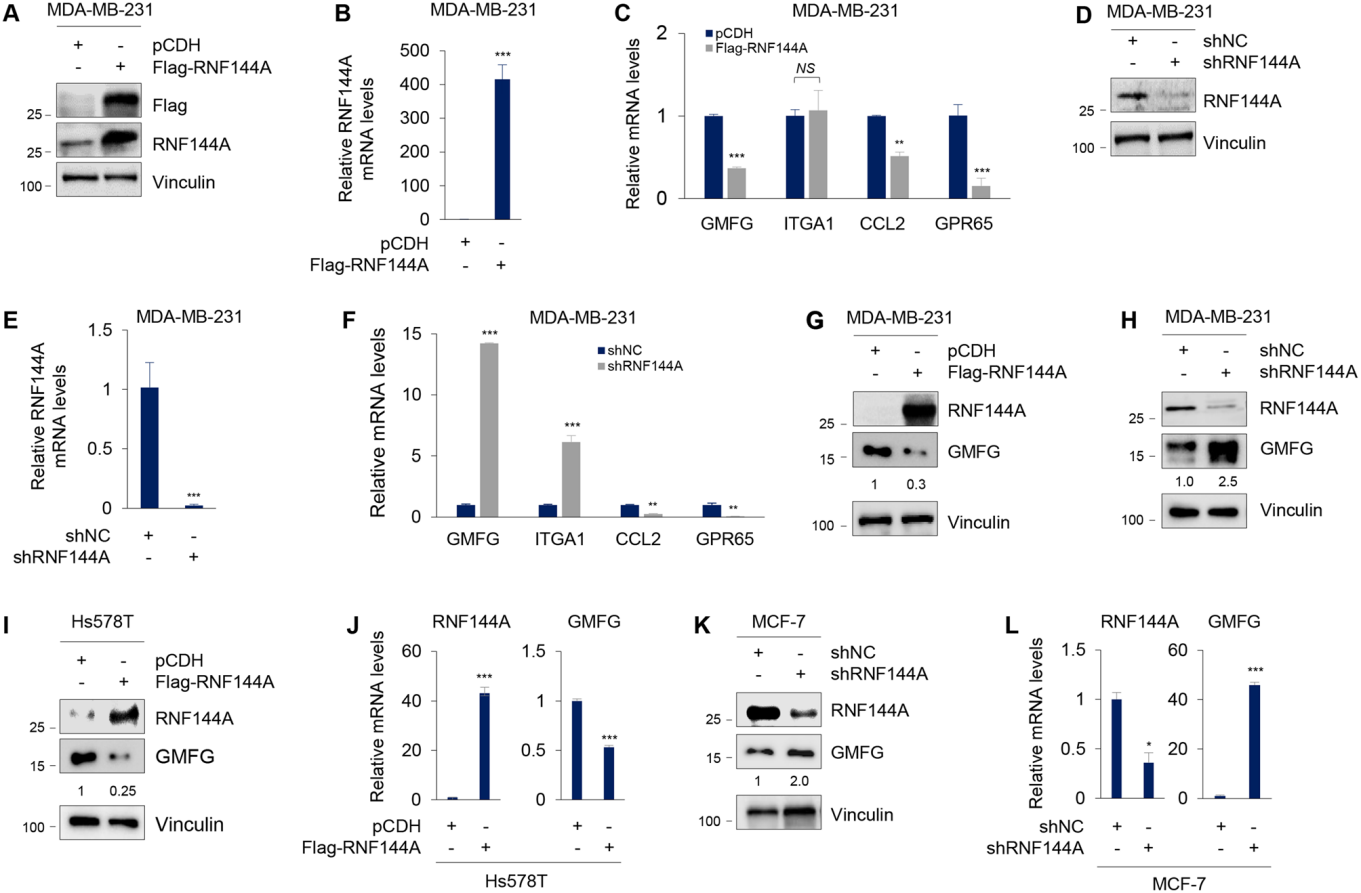
RNF144A negatively regulates GMFG expression in breast cancer cells. **A** and **B** MDA-MB-231 cells stably expressing pCDH and Flag-RNF144A were subjected to immunoblotting (**A**) and qPCR (**B**) analysis of RNF144A expression levels. **C** Validation of differentially expression genes by qPCR in MDA-MB-231 cells stably expressing pCDH and Flag-RNF144A. **D** and **E** MDA-MB-231 cells stably expressing shNC and shRNF44A were subjected to immunoblotting (**D**) and qPCR (**E**) analysis of RNF144A expression levels. **F** Validation of differentially expression genes by qPCR in MDA-MB-231 cells stably expressing shNC and shRNF144A. **G** MDA-MB-231 cells stably expressing pCDH and Flag-RNF144A were subjected to immunoblotting analysis with the indicated antibodies. **H** MDA-MB-231 cells stably expressing shNC and shRNF144A were subjected to immunoblotting analysis with the indicated antibodies. **I** and **J** Hs578T cells stably expressing pCDH and Flag-RNF144A were subjected to immunoblotting (**I**) and qPCR (**J**) analysis of RNF144A and GMFG expression levels. **K** and **L** MCF-7 cells stably expressing shNC and shRNF144A were subjected to immunoblotting (**K**) and qPCR (**L**) analysis of RNF144A and GMFG expression levels. *NS* no significance; *p < 0.05; ** *p* < 0.01; *** *p* < 0.001

According to the above analysis, 4 genes with definite functions in human cancer, including GMFG, integrin subunit α 1 (ITGA1), C–C motif chemokine ligand 2 (CCL2) and G Protein-coupled receptor 65 (GPR65) were selected for further validation by qPCR. Results showed that the mRNA levels of GMFG, CCL2, and GPR65 were decreased in MDA-MB-231 cells stably expressing Flag-RNF144A compared to cells expressing empty vector pCDH (Fig. 2A–C). In contrast, there were no significant difference in the mRNA levels of ITGA1 between pCDH- and Flag-RNF144A-expressing cells (Fig. 2C). In addition, the mRNA levels of GMFG and ITGA1 were increased, whereas the mRNA levels of CCL2 and GPR65 were decreased, in MDA-MB-231 cells stably expressing shRNF144A relative to cells expressing shNC (Fig. 2D–F). Based on these results, we therefore subsequently focused on addressing the regulatory mechanism of GMFG by RNF144A. Immunoblotting analysis showed that ectopic expression of RNF144A in MDA-MB-231 cells downregulated (Fig. 2G), whereas knockdown of endogenous RNF144A in MDA-MB-231 cells upregulated (Fig. 2H), the protein levels of GMFG. Similar results were also obtained in RNF144A-overexpressing Hs578T cells (Fig. 2I, J) and RNF144A-depleted MCF-7 cells (Fig. 2K, L). These results suggest that RNF144A negatively regulates GMFG expression in breast cancer cells.

### RNF144A suppresses breast cancer cell proliferation, migration, and invasion through, at least in part, regulating GMFG expression

**Fig. 3:**
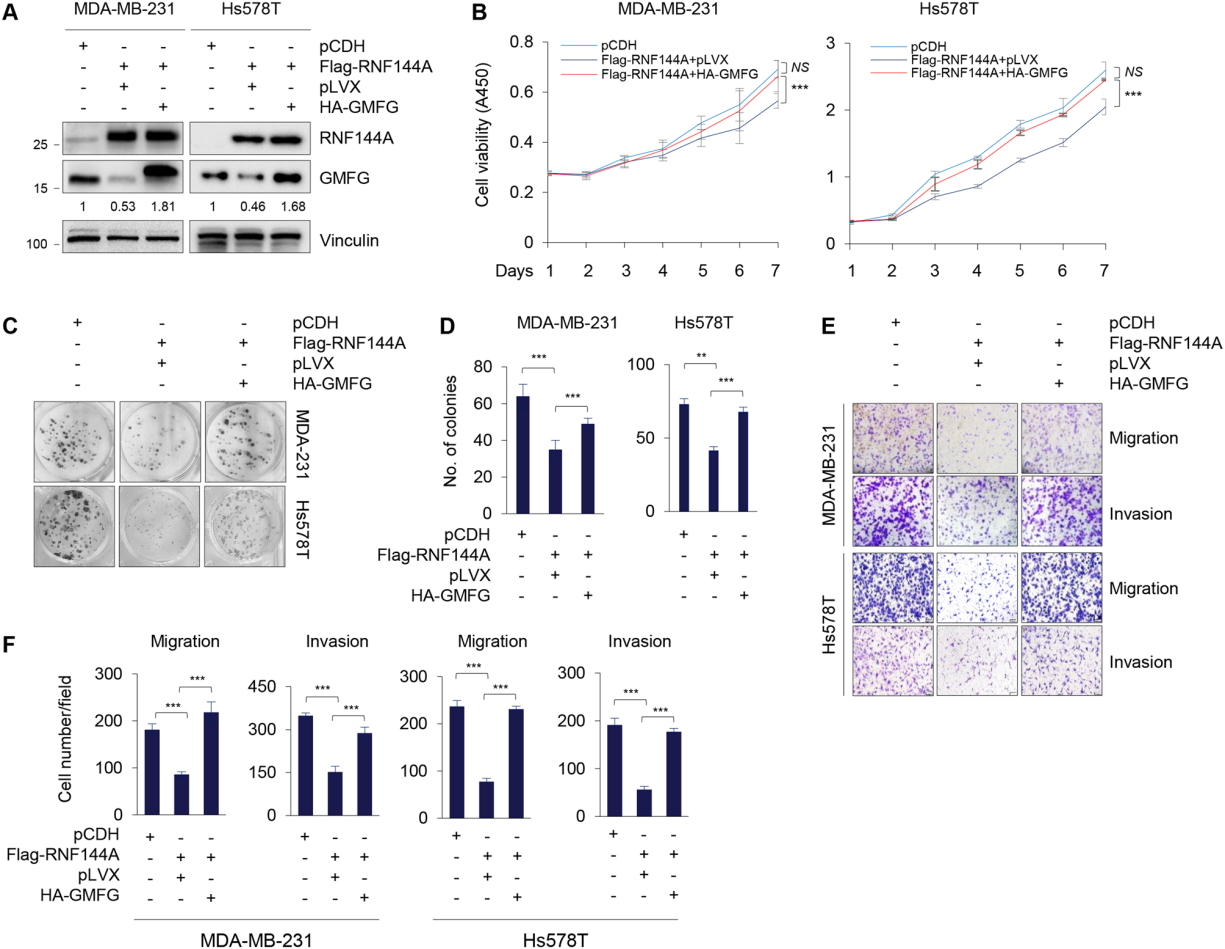
RNF144A suppresses breast cancer cell proliferation, migration, and invasion partially through regulating GMFG expression. **A** Establishment of stable MDA-MB-231 and Hs578T cell lines expressing Flag-RNF144A alone or in combination with HA-GMFG by lentiviral infection. Expression levels of RNF144A and GMFG in the established cell lines were verified by immunoblot analysis with the indicated antibodies. **B**, **C**, and **D** MDA-MB-231 and Hs578T cell lines expressing Flag-RNF144A alone or in combination with HA-GMFG were subjected to CCK-8 (B) and colony-formation assays (**C**, **D**). The representative survival colonies and corresponding quantitative results are shown in **C** and **D**, respectively. **E** and **F** MDA-MB-231 and Hs578T cell lines expressing Flag-RNF144A alone or in combination with HA-GMFG were subjected transwell migration and invasion assays. The representative images of migrated and invaded cells and corresponding quantitative results are shown in E and F, respectively. ** *p* < 0.01; *** *p* < 0.001

To address whether RNF144A acts as a tumor suppressor in breast cancer through regulating GMFG expression, we re-expressed GMFG in MDA-MB-231 and Hs578T cells stably expressing RNF144A (Fig. 3A). CCK8 and colony formation assays revealed that ectopic expression of RNF144A in MDA-MB-231 and Hs578T cells suppressed breast cancer cell proliferation (Fig. 3B) and colony formation (Fig. 3C, D), but the noted effects were partially rescued following re-expression of GMFG in RNF144A overexpressing cells. In addition, transwell migration and invasion assays showed that suppression of migratory and invasive potential of MDA-MB-231 and Hs578T cells by ectopic expression of RNF144A was partially restored by re-expression of GMFG in cells stably expressing RNF144A (Fig. 3E, F). Collectively, these results suggest that RNF144A suppresses breast cancer cell proliferation, migration, and invasion through, at least in part, regulating GMFG expression.

### Transcription factor YY1 positively regulates GMFG expression

**Fig. 4:**
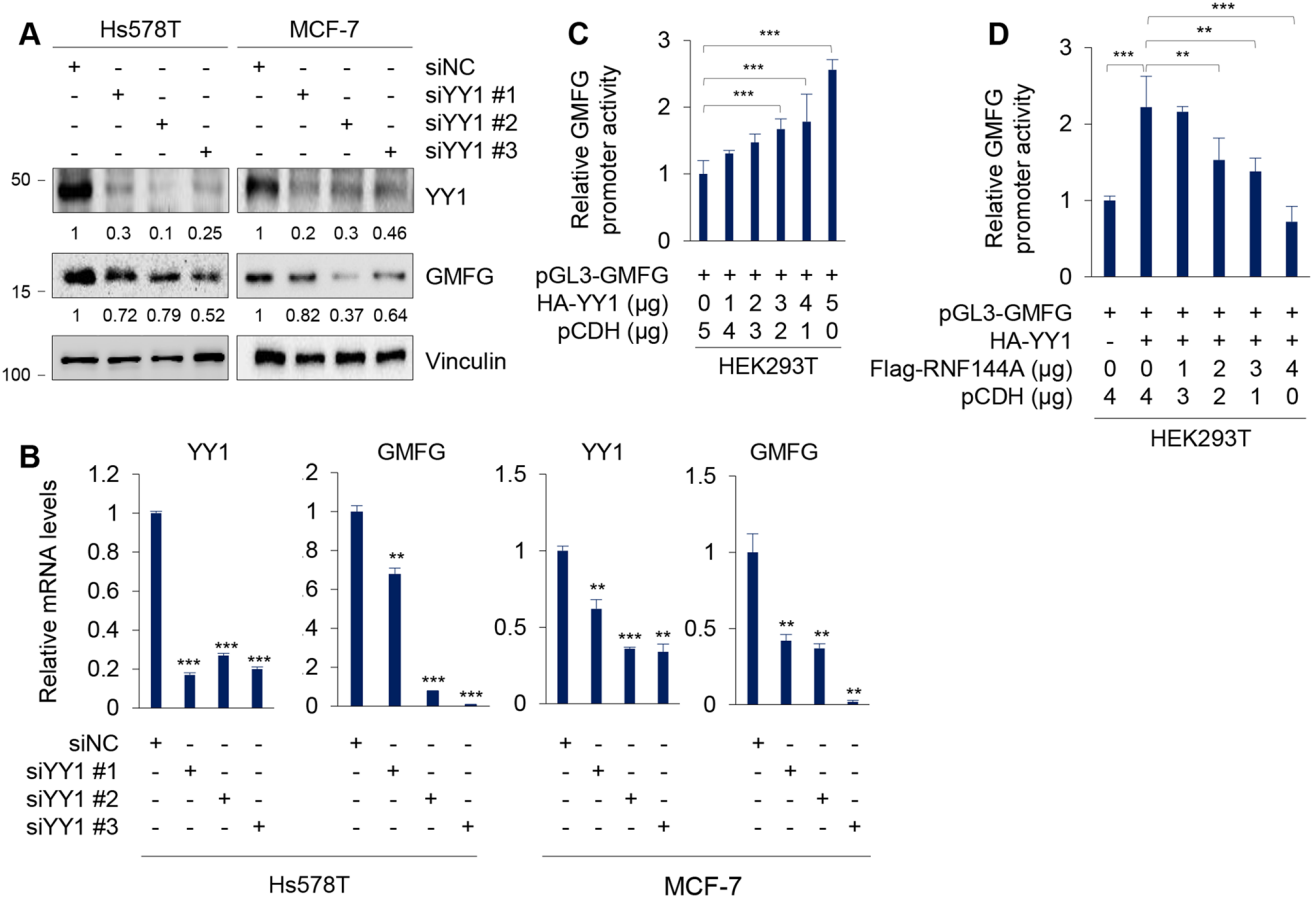
YY1 transcriptionally activates GMFG expression. **A** and **B** Hs578T and MCF-7 cells were transfected with three independent siRNAs targeting YY1 (siYY #1–3) and control siRNA (siNC). After 48 h of transfection, cells were collected for immunoblotting (**A**) and qPCR (**B**) analysis of the expression levels of YY1 and GMFG. **C** HEK293T cells were transfected with pGL3-GMFG alone or increasing doses of HA-YY1. After 48 h of transfection, the promoter activity of GMFG was detected by luciferase assays. **D** HEK293T cells were transfected with pGL3-GMFG, HA-YY1, or in combination with increasing doses of Flag-RNF144A. After 48 h of transfection, the promoter activity of GMFG promoter was detected by luciferase assays. ** *p* < 0.01; *** *p* < 0.001

As RNF144A is not a putative transcription factor, we next examined potential transcription factors that bind to the promoter of GMFG using UCSC Genome Browser (https:// genome.ucsc.edu) [44] and JASPAR (http://jaspar.genereg.net/) [45] programs, and identified transcription factor YY1 could regulate GMFG expression. To verify this notion, we transfected Hs578T and MCF-7 cells with three independent siRNAs targeting YY1 (siYY1 #1-3). Immunoblotting and qPCR assays showed that knockdown of YY1 reduced the protein and mRNA levels of GMFG in both cell lines (Fig. 4A, B). Luciferase reporter assays demonstrated that YY1 enhanced the promoter activity of GMFG in a dose-dependent manner (Fig. 4C). Moreover, co-transfection of RNF144A attenuated YY1-induced increase in GMFG promoter activity (Fig. 4D). These results suggest that transcription factor YY1 positively regulates GMFG expression, and this process is blocked in the presence of RNF144A.

### RNF144A negatively regulates the stability of YY1

**Fig. 5:**
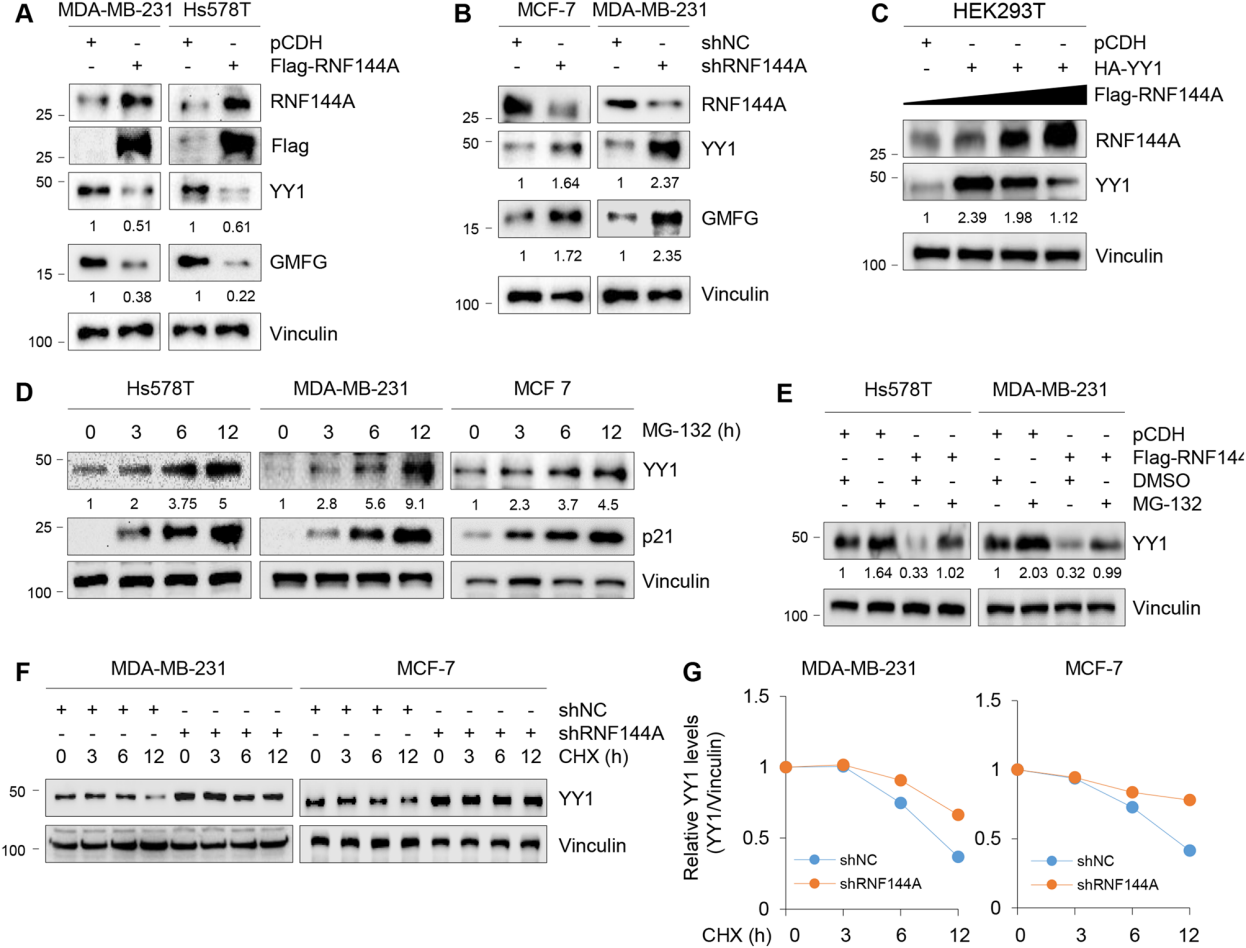
RNF144A negatively affects the stability of YY1 protein. **A** MDA-MB-231 and Hs578T cells stably expressing pCDH and Flag-RNF144A were subjected to immunoblotting analysis with the indicated antibodies. **B** MCF-7 and MDA-MB-231 cells stably expressing shNC and shRNF144A were subjected to immunoblotting analysis with the indicated antibodies. **C** HEK293T cells were transfected with HA-YY1 alone or in combination with increasing doses of Flag-RNF144A. After 48 h of transfection, cells were collected for immunoblotting analysis with the indicated antibodies. **D** Hs578T, MDA-MB-231, and MCF-7 cells were treated with DMSO or 10 μM of MG-132 for the indicated times, and then subjected to immunoblotting analysis with the indicated antibodies. **E** MDA-MB-231 and Hs578T cells stably expressing pCDH and Flag-RNF144A were treated with DMSO or 10 μM of MG-132 for 6 h, and then subjected to immunoblotting analysis with the indicated antibodies. **F** and **G** MDA-MB-231 and MCF-7 cell lines stably expressing shNC and shRNF144A were treated with DMSO or 100 μg/ml of CHX for the indicated times, and then subjected to immunoblotting analysis with the indicated antibodies. The representative images of immunoblotting and corresponding quantitative results (YY1/Vinculin) are shown in F and G, respectively

To address the molecular mechanism by which RNF144A blocks YY1-induced GMFG expression, we next examined whether RNF144A affects the stability of YY1. As shown in Fig. 5A, ectopic expression of RNF144A in MDA-MB-231 and Hs578T cells resulted in a decrease in the protein levels of YY1 and GMFG. In contrast, knockdown of endogenous RNF144A in MCF-7 and MDA-MB-231 cells upregulated the protein levels of YY1 and GMFG (Fig. 5B). Moreover, ectopic expression of Flag-RNF144A in HEK293T cells led to a reduction in the protein levels of HA-YY1 in a dose-dependent manner (Fig. 5C). To verify whether YY1 can be degraded through ubiquitin–proteasome pathway in breast cancer cells, MDA-MB-231, Hs578T, and MCF-7 cells were treated with proteosome inhibitor MG-132 for the indicated times. Immunoblotting analysis showed that the protein levels of YY1 were increased after MG-132 treatment in a time-dependent manner (Fig. 5D). As a positive control, MG-132 treatment resulted in a significant increase in the protein levels of p21, a known substrate of the ubiquitin–proteasome system [46]. Moreover, RNF144A-mediated downregulation of YY1 in Hs578T and MDA-MB-231 cells was restored following MG-132 treatment (Fig. 5E). Knockdown of RNF144A in MDA-MB-231 and MCF-7 cells extended the half-life of YY1 protein (Fig. 5F, G). Collectively, these results suggest that RNF144A negatively regulates the stability of YY1.

### RNF144A interacts with YY1 and promotes its ubiquitination

**Fig. 6:**
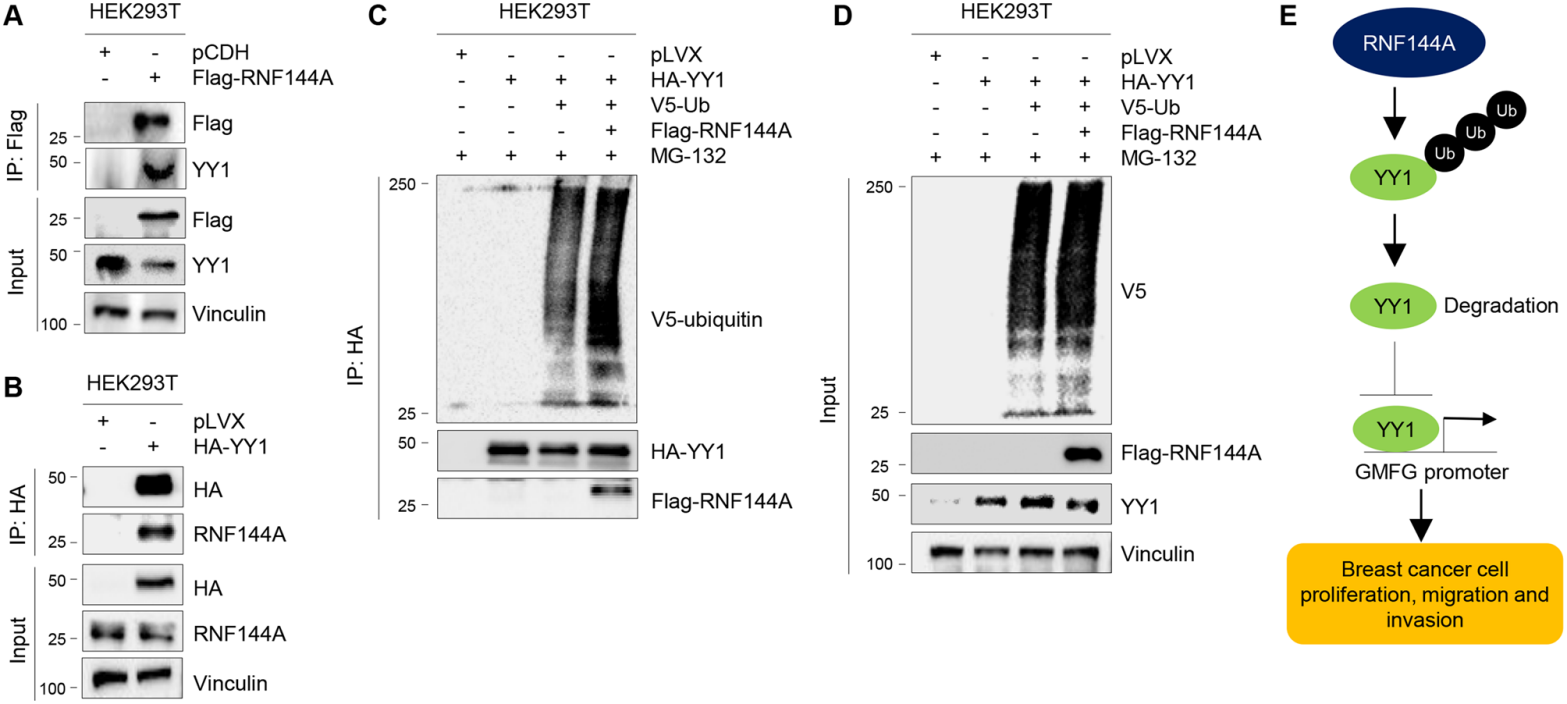
RNF144A interacts with YY1 and promotes its ubiquitination. **A** HEK293T cells were transfected with empty vector pCDH and Flag-RNF144A. After 48 of transfection, total cellular lysates were subjected to IP and immunoblotting analysis with the indicated antibodies. **B** HEK293T cells were transfected with empty vector pLVX and HA-YY1. After 48 of transfection, total cellular lysates were subjected to IP and immunoblotting analysis with the indicated antibodies. **C** and **D** HEK293T cells were transfected with HA-YY1, V5-ubiquitin alone or in combination with Flag-RNF144A. After 48 h of transfection, cells were treated with 10 μM MG-132 for 6 h, and then subjected to sequential IP and immunoblotting analysis with the indicated antibodies. **E** The proposed working model. Transcription factor YY1 transcriptionally activates GMFG expression to promote breast cancer cell proliferation, migration, and invasion. RNF144A interacts with YY1 and promotes its proteasomal degradation, thus blocking YY1-induced GMFG expression and suppressing breast cancer cell proliferation, migration, and invasion

To address how RNF144A regulates the stability of YY1, we next examined whether RNF144A interacts with YY1. To this aim, HEK293T cells were transfected with Flag-RNF144A or HA-YY1, then subjected to immunoprecipitation assays with either an anti-Flag or anti-HA antibody. Immunoblotting analysis showed that Flag-RNF144A interacted with endogenous YY1 in HEK293T cells (Fig. 6A), while HA-YY1 was co-immunoprecipiated with endogenous RNF144A (Fig. 6B), indicating that RNF144A and YY1 can interact with each other in vivo. To examine whether RNF144A regulates YY1 ubiquitination levels, HEK293T cells were transfected with HA-YY1, V5-ubiquitin, Flag-RNF144A alone or in combination. As shown in Fig. 6C, D, ectopic expression of Flag-RNF144A led to an increase in the ubiquitination levels of HA-YY1. These results suggest that RNF144A promotes the proteasomal degradation of YY1 by enhancing its polyubiquitination (Fig. 6E).

## Discussion

Our previous studies showed that RNF144A is epigenetically silenced in breast cancer cells by promoter hypermethylation [15] and its expression levels are associated with favorable prognosis of breast cancer patients [16]. In this study, we provide new mechanistic insights into tumor suppressor function of RNF144A in breast cancer cells.

First, RNF144A targets transcription factor YY1 for proteasomal degradation. Accumulating evidence shows that YY1 is overexpressed in multiple cancer types and that increased YY1 levels correlate with poor clinical outcomes [47–49]. In breast cancer, YY1 functions as an oncogene and negatively regulates p27 expression [28]. Previous studies have demonstrated that E3 ubiquitin ligase Smurf2 regulates proteasomal degradation of YY1 in multiple model systems [40–43]. In addition, E3 ubiquitin ligase TRIP12 interacts with and ubiquitinates YY1, leading to its degradation [50]. In contrast, ubiquitin specific protease 21 (USP21) stabilizes YY1 in non-small-cell lung cancer cells via mediating its deubiquitination [51]. E3 SUMO-protein ligase PIAS4 stimulates the sumoylation of YY1 and stabilizes YY1 [52]. In this study, we demonstrated that E3-ubiquitin ligase RNF144A interacts with YY1 and promotes its proteosomal degradation through ubiquitination-dependent pathway (Figs. 5 and 6). As RNF144A is downregulated in breast cancer cell lines and tumor tissues [15, 16], which could be partially contribute to overexpression of YY1 in breast cancer cells [28].

Second, YY1 transcriptionally activates GMFG expression, and this process is blocked by RNF144A. Transcription factor YY1 can activate or inactivate gene expression depending on interacting partners, promoter context and chromatin structure, and may be involved in the transcriptional control of about 10% of the total mammalian gene set [47, 53]. In our study, we found that knockdown of YY1 results in a decrease in the protein and mRNA levels of GMFG. Moreover, ectopic expression of YY1 enhances the promoter activity of GMFG in a dose-dependent manner, whereas RNF144A significantly suppresses YY1-induced increase in the promoter activity of GMFG gene (Fig. 4). Previous studies have shown that high GMFG expression correlates with poor prognosis and promotes cell migration and invasion in epithelial ovarian cancer [25]. In addition, expression of GMFG is associated with colorectal cancer metastasis and its downregulation suppresses colorectal cancer cell migration and invasion [24]. We further demonstrated that RNF144A suppresses breast cancer cell growth, migration, and invasion through, at least in part, negatively regulating GMFG expression (Fig. 3).

In conclusion, the findings presented in this study suggest that RNF144A exerts tumor suppressor functions in breast cancer cells through targeting transcription factor YY1 for proteasomal degradation, thus blocking YY1-mediated transcriptional activation of GMFG expression in breast cancer cells. These results expand our current understanding of the mechanistic roles of RNF144A in breast cancer progression and lay a foundation for further exploring its clinical application to impede cancer development and progression or sensitize cancer cells to anticancer drugs.

## Supplementary Information

Below is the link to the electronic supplementary material.

Supplementary file1 (DOCX 42 kb)

## Data Availability

All data generated or analyzed during this study are included in this manuscript.

## Abbreviations

GMFG: Glia maturation factor gamma
RNF144A: Ring finger protein 144A
YY1: Yin Yang 1
