# Supplementary material for: RNF144A exerts tumor suppressor function in breast cancer through targeting YY1 for proteasomal degradation to downregulate GMFG expression
Source: Med Oncol. 2022 Feb 1;39(4):48. doi: 10.1007/s12032-021-01631-6 (PMC8807444; doi:10.1007/s12032-021-01631-6)
Supplement: Supplementary file 1 — Supplementary file1 (DOCX 42 kb) [file 12032_2021_1631_MOESM1_ESM.docx]

**Supplementary information for**

Zhang et al. RNF144A exerts tumor suppressor function in breast cancer through targeting YY1 for proteasomal degradation to downregulate GMFG expression

**The Supporting information contains:**

Supplementary Tables S1-S4

**Supplementary Tables**

**Table S1. Primers used for molecular cloning analysis**

| Plasmids | Primers | Sequences |
| --- | --- | --- |
| HA-YY1 | Forward | GGATCTATTTCCGGTGAATTCGCCACCATGGCCTCGGGCGACAC |
|  | Reverse | GGGATCCGCGGCCGCTCTAGATTAAGCGTAGTCTGGGACGTCGT |
| HA-GMFG | Forward | TTCCTCGAGACTAGTTCTGCCATGTCTGACTCCCTGGTGGTG |
|  | Reverse | GGATCCGCGGCCGCTCTTTAAGCGTAGTCTGGGACGTCGTATGGGTATACGAAAGAAAGACAACT |

**Table S2. siRNA target sequences**

| siRNAs | Sequences |
| --- | --- |
| siYY1 #1 | CCUCCUGAUUAUUCAGAAUTT |
| siYY1 #2 | CUGGCAGAAUUUGCUAGAATT |
| siYY1 #3 | CAGUCAACUAACCUGAAAUTT |

**Table S3. Primers for qPCR analysis**

| Genes |  | Primers (5’-3’) |
| --- | --- | --- |
| RNF144A | Forward | CCACCTACAGGAGAACGAG |
|  | Reverse | TCCGACAGGGATCAAACA |
| GMFG | Forward | GGAAATTCCGCTTCCGAAAAGA |
|  | Reverse | GTAGCTGTAAACCACGAACCTG |
| ITGA1 | Forward | GCTCCTCACTGTTGTTCTACG |
|  | Reverse | CGGGCCGCTGAAAGTCATT |
| CCL2 | Forward | CAGCCAGATGCAATCAATGCC |
|  | Reverse | TGGAATCCTGAACCCACTTCT |
| GPR65 | Forward | GCATTGCCGTTGATCGGTATT |
|  | Reverse | CGTCCTGAACAAGTTGAGGTT |
| YY1 | Forward | ACGGCTTCGAGGATCAGATTC |
|  | Reverse | TGACCAGCGTTTGTTCAATGT |
| GAPDH | Forward | CGAGATCCCTCCAAAATCAA |
|  | Reverse | TTCACACCCATGACGAACAT |

**Table S4. 128 differentially expressed genes between pCDH- and RNF144A-expressing MDA-MB-231 cells with fold change over 1.5**

|  | Gene Symbol | Regulation | Fold change (abs) | *P* value |
| --- | --- | --- | --- | --- |
| 1 | MIR2054 | Up | 5.6699066 | 0.025165452 |
| 2 | RNF144A | Up | 3.31961 | 2.66E-04 |
| 3 | LOC100507056 | Up | 2.257051 | 0.01562504 |
| 4 | ZNF404 | Up | 1.9382963 | 0.005334039 |
| 5 | LOC647859 | Up | 1.9300497 | 3.57E-04 |
| 6 | AGR2 | Up | 1.8991559 | 4.66E-04 |
| 7 | OCIAD1-AS1 | Up | 1.8648851 | 0.004400178 |
| 8 | LAMC2 | Up | 1.8063004 | 2.14E-04 |
| 9 | TAS2R50 | Up | 1.7729917 | 0.00844538 |
| 10 | NTN4 | Up | 1.7033168 | 0.002465762 |
| 11 | OCLN | Up | 1.6811315 | 6.84E-04 |
| 12 | TNS4 | Up | 1.670021 | 1.67E-06 |
| 13 | CST4 | Up | 1.6580061 | 0.010601806 |
| 14 | IGHD3-3 | Up | 1.6396176 | 0.022269737 |
| 15 | TMEM156 | Up | 1.6307449 | 2.14E-04 |
| 16 | SLAMF7 | Up | 1.6243253 | 0.002818842 |
| 17 | MYO5C | Up | 1.6178881 | 7.84E-05 |
| 18 | TMEM154 | Up | 1.6142688 | 0.002212423 |
| 19 | TMEM45B | Up | 1.6010835 | 5.35E-04 |
| 20 | OR5H6 | Up | 1.5915393 | 0.016207054 |
| 21 | TBC1D8 | Up | 1.5863189 | 8.58E-04 |
| 22 | KRT81 | Up | 1.576456 | 7.48E-04 |
| 23 | LOC100216001 | Up | 1.5736542 | 8.84E-04 |
| 24 | VSIG1 | Up | 1.559807 | 0.003788157 |
| 25 | KYNU | Up | 1.5595115 | 8.31E-04 |
| 26 | SRGAP1 | Up | 1.5549493 | 0.00230162 |
| 27 | ARSJ | Up | 1.5436449 | 5.01E-04 |
| 28 | LOC646627 | Up | 1.5436071 | 5.13E-04 |
| 29 | SPANXB1 | Up | 1.540719 | 0.003425248 |
| 30 | SPANXB1 | Up | 1.540719 | 0.003425248 |
| 31 | CST2 | Up | 1.5365957 | 0.008051318 |
| 32 | PLCE1 | Up | 1.5356255 | 3.91E-05 |
| 33 | SAMD9 | Up | 1.5342664 | 2.32E-04 |
| 34 | FOXQ1 | Up | 1.5282042 | 0.00180565 |
| 35 | LINC00261 | Up | 1.525467 | 0.00284212 |
| 36 | SEMA3A | Up | 1.5238425 | 8.76E-04 |
| 37 | ATP6V0D2 | Up | 1.5232301 | 0.007000155 |
| 38 | RAB3B | Up | 1.5232024 | 8.67E-04 |
| 39 | TAS2R43 | Up | 1.5165021 | 0.011950178 |
| 40 | AFAP1-AS1 | Up | 1.5141897 | 0.001678075 |
| 41 | PELI2 | Up | 1.5104749 | 0.011247567 |
| 42 | JAG1 | Up | 1.5058998 | 5.52E-04 |
| 43 | TMC7 | Up | 1.5004561 | 2.87E-04 |
| 44 | SNORD123 | Down | 2.7167435 | 0.003043621 |
| 45 | GPR65 | Down | 2.621815 | 0.002352519 |
| 46 | CDK15 | Down | 2.6167054 | 3.25E-04 |
| 47 | SORCS2 | Down | 2.5866096 | 4.68E-06 |
| 48 | CST7 | Down | 2.429816 | 5.71E-04 |
| 49 | MAMDC2 | Down | 2.311691 | 8.54E-04 |
| 50 | HLA-DPA1 | Down | 2.3051775 | 3.48E-04 |
| 51 | CYP24A1 | Down | 2.2480655 | 8.69E-04 |
| 52 | CCNA1 | Down | 2.2397175 | 3.25E-04 |
| 53 | HLA-DRA | Down | 2.208082 | 4.31E-04 |
| 54 | KAL1 | Down | 2.1711547 | 1.39E-04 |
| 55 | TNFSF10 | Down | 2.165068 | 1.21E-04 |
| 56 | CCL2 | Down | 2.1379309 | 0.011995583 |
| 57 | GMFG | Down | 2.0898817 | 1.10E-05 |
| 58 | MYH10 | Down | 2.0895073 | 6.41E-05 |
| 59 | MGC12916 | Down | 2.0579696 | 0.004064032 |
| 60 | RNF125 | Down | 2.0136292 | 3.77E-04 |
| 61 | TGFBI | Down | 2.0002894 | 5.62E-06 |
| 62 | CFB | Down | 1.9858178 | 0.001660467 |
| 63 | NNMT | Down | 1.9798343 | 5.08E-05 |
| 64 | PAPPA | Down | 1.9357765 | 1.06E-04 |
| 65 | MATN2 | Down | 1.9128165 | 1.56E-04 |
| 66 | ITGA1 | Down | 1.911569 | 0.004641861 |
| 67 | KIAA1199 | Down | 1.8983344 | 3.10E-04 |
| 68 | ADAMTS9 | Down | 1.893707 | 2.81E-04 |
| 69 | ADAMTS12 | Down | 1.8813907 | 3.32E-05 |
| 70 | HLA-DRB1 | Down | 1.848046 | 0.00309441 |
| 71 | LOC100505806 | Down | 1.8448048 | 8.05E-04 |
| 72 | LPCAT2 | Down | 1.8290684 | 0.001772468 |
| 73 | IGHV3-15 | Down | 1.825826 | 0.004926338 |
| 74 | CALB2 | Down | 1.8213406 | 2.43E-04 |
| 75 | EDNRB | Down | 1.8149521 | 0.001383266 |
| 76 | GBP4 | Down | 1.8121834 | 8.55E-05 |
| 77 | FLG | Down | 1.8078903 | 0.003565156 |
| 78 | PDE7B | Down | 1.7906148 | 8.49E-05 |
| 79 | COL12A1 | Down | 1.7754517 | 7.43E-04 |
| 80 | CLIC5 | Down | 1.7707468 | 2.61E-05 |
| 81 | CCBE1 | Down | 1.7693896 | 0.001291325 |
| 82 | HAS2 | Down | 1.7689066 | 4.00E-04 |
| 83 | GNAO1 | Down | 1.7632661 | 0.002299408 |
| 84 | C3 | Down | 1.7608328 | 0.00124239 |
| 85 | COL6A3 | Down | 1.7585698 | 7.79E-05 |
| 86 | RGS4 | Down | 1.7387108 | 0.014635292 |
| 87 | LRRC61 | Down | 1.7155591 | 9.71E-04 |
| 88 | GPR85 | Down | 1.7104726 | 1.36E-04 |
| 89 | KCNIP3 | Down | 1.7103578 | 9.69E-04 |
| 90 | FN1 | Down | 1.7093298 | 0.001417518 |
| 91 | HLA-DRB5 | Down | 1.6943727 | 0.002463172 |
| 92 | ZNF175 | Down | 1.6900017 | 0.03875292 |
| 93 | IRX3 | Down | 1.6896052 | 7.59E-04 |
| 94 | CXADR | Down | 1.6854193 | 6.45E-04 |
| 95 | LY6E | Down | 1.6845193 | 0.001072192 |
| 96 | LGR5 | Down | 1.6749862 | 1.61E-05 |
| 97 | RARRES3 | Down | 1.6634918 | 0.001906695 |
| 98 | SAA2 | Down | 1.6622475 | 9.77E-04 |
| 99 | PARD6B | Down | 1.6606748 | 2.25E-04 |
| 100 | LOX | Down | 1.6420467 | 1.30E-04 |
| 101 | RN5S464 | Down | 1.6351482 | 0.012633268 |
| 102 | SLIT2 | Down | 1.6319069 | 5.62E-04 |
| 103 | TGM2 | Down | 1.629746 | 6.73E-06 |
| 104 | HS3ST3B1 | Down | 1.627455 | 5.72E-04 |
| 105 | SAA1 | Down | 1.6262462 | 0.004886331 |
| 106 | ARHGAP15 | Down | 1.6061234 | 8.40E-04 |
| 107 | TTC39C | Down | 1.6030867 | 0.001659305 |
| 108 | IL8 | Down | 1.6013142 | 9.64E-04 |
| 109 | SERPINA1 | Down | 1.5996802 | 2.12E-04 |
| 110 | C7orf29 | Down | 1.5936773 | 0.004261133 |
| 111 | PTGS2 | Down | 1.5846362 | 0.009792233 |
| 112 | CD82 | Down | 1.566295 | 5.94E-04 |
| 113 | NIPAL1 | Down | 1.5565387 | 0.001229453 |
| 114 | NEDD4L | Down | 1.5544108 | 8.49E-05 |
| 115 | LRIG1 | Down | 1.5540429 | 4.66E-04 |
| 116 | L1CAM | Down | 1.5399085 | 1.16E-04 |
| 117 | M ARCH4 | Down | 1.5316033 | 0.024183571 |
| 118 | CABLES1 | Down | 1.53138 | 2.38E-04 |
| 119 | HLA-B | Down | 1.5307369 | 4.67E-05 |
| 120 | EGR1 | Down | 1.5294322 | 0.00126321 |
| 121 | INE1 | Down | 1.5289409 | 0.036766008 |
| 122 | CXCL1 | Down | 1.5237275 | 6.91E-05 |
| 123 | SDPR | Down | 1.5189341 | 0.0034202 |
| 124 | IGHJ5 | Down | 1.5148184 | 0.016790451 |
| 125 | HLA-DPB1 | Down | 1.5074754 | 5.88E-04 |
| 126 | BICC1 | Down | 1.5072004 | 4.49E-05 |
| 127 | LIPG | Down | 1.5063717 | 1.30E-05 |
| 128 | CPNE2 | Down | 1.5006579 | 1.67E-04 |
